# Face mediated human–robot interaction for remote medical examination

**DOI:** 10.1038/s41598-022-16643-z

**Published:** 2022-07-22

**Authors:** Thilina D. Lalitharatne, Leone Costi, Ryman Hashem, Ilana Nisky, Rachael E. Jack, Thrishantha Nanayakkara, Fumiya Iida

**Affiliations:** 1 Department of Engineering, University of Cambridge, Cambridge, UK; 2 Dyson School of Design Engineering, Imperial College London, London, UK; 3 Department of Biomedical Engineering, Ben-Gurion University of the Negev, Beersheba, Israel; 4 School of Psychology and Neuroscience, University of Glasgow, Glasgow, UK

**Keywords:** Mechanical engineering, Biomedical engineering

## Abstract

Realtime visual feedback from consequences of actions is useful for future safety-critical human–robot interaction applications such as remote physical examination of patients. Given multiple formats to present visual feedback, using face as feedback for mediating human–robot interaction in remote examination remains understudied. Here we describe a face mediated human–robot interaction approach for remote palpation. It builds upon a robodoctor–robopatient platform where user can palpate on the robopatient to remotely control the robodoctor to diagnose a patient. A tactile sensor array mounted on the end effector of the robodoctor measures the haptic response of the patient under diagnosis and transfers it to the robopatient to render pain facial expressions in response to palpation forces. We compare this approach against a direct presentation of tactile sensor data in a visual tactile map. As feedback, the former has the advantage of recruiting advanced human capabilities to decode expressions on a human face whereas the later has the advantage of being able to present details such as intensity and spatial information of palpation. In a user study, we compare these two approaches in a teleoperated palpation task to find the hard nodule embedded in the remote abdominal phantom. We show that the face mediated human–robot interaction approach leads to statistically significant improvements in localizing the hard nodule without compromising the nodule position estimation time. We highlight the inherent power of facial expressions as communicative signals to enhance the utility and effectiveness of human–robot interaction in remote medical examinations.

## Introduction

The human face is one of the most powerful and efficient interfaces used to communicate the myriad complex messages that support human interactions. For example, a face can produce about twenty thousand different facial expressions^1^. The anatomy of the human face reveals that it consists of many independent muscles which can be voluntarily controlled to generate complex and visually salient dynamic facial expressions^2^. However, the effectiveness of the face in communicating simple physical states, such as the indentation level on a painful area of the abdomen during palpation, compared to the direct visualization of a stress map caused by the indentation has not been reported before. The facial expressions can effectively communicate such pain information^3^ is known insofar as physicians palpate real people and look at their facial expressions to make inferences about potential sources of pain. Information extracted from the face mainly depends on three related components: facial dynamics, morphology, and complexion^4^. Thus, given that the human face is a complex dynamic visual stimulus, it can transfer complex information between individuals to support myriad interactions.

Commensurate with the complexity of the face, humans can decode facial information quickly and efficiently using a highly co-evolved visual system that is tuned to detect these features, such as the high contrast of the eye whites and high spatial frequencies of the wrinkled nose^5^. To accurately decoding the incoming facial expression signal, receivers must associate the detected facial features with their prior knowledge of it’s meaning in a given culture or context^4^. Thus, the human brain has co-evolved to efficiently decode facial expression signals to support adaptive action^6^.

Accurately decoding facial expressions is a critical skill in everyday human communication^7^, and especially in the medical field as patients often communicate pain and emotions using facial expressions^8,9^ particularly during physical examinations. Specifically, physicians palpate an area such as the abdomen to detect tenderness or masses^10^ as well as anomalies in the internal organs^11^. The combination of haptic and visual feedback during such physical examinations are routinely used in successfully diagnosing physiological conditions in patients. For example, visual feedback via patient facial expressions during manual palpation is often used to assess a range of medical hypotheses for diagnosis.

**Figure 1:**
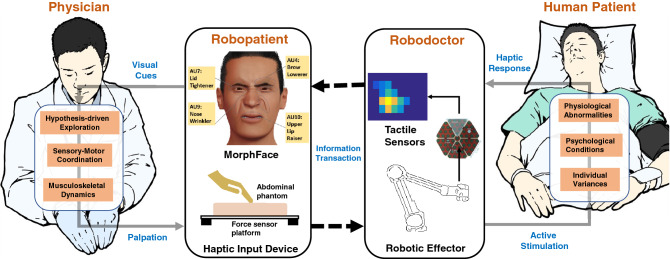
Face mediated human–robot interaction approach for remote palpation. The system consists of four main subsystems: a physician, a robopatient, a robodoctor, and a human patient. The human patient under examination may have various physiological abnormalities, psychological conditions, and individual characteristics that vary these factors. To diagnose the conditions of the human patient remotely, the physician at the local site interacts with the roboatient via a haptic input device which consists of a force sensor platform and an abdominal phantom. The acquired haptic information is then transferred to the robodoctor that stimulates the human patient using a robotic effector. The active stimulation provided by the robotic effector causes the patient to generate haptic responses which are measured by the tactile sensors mounted on the robotic effector. High-dimensional tactile information acquired by the robotdoctor is then transferred to the robotpatient where the high-dimensional information is encoded into a low-dimensional representation by synthesizing facial expressions using our hybrid morphable robotic face: MorphFace^12^. Four facial Action Units (AUs) namely, AU4: Brow Lowerer, AU7: Lid Tightener, AU9: Nose Wrinkler, AU10: Upper Lip Raiser (based on Ekmans facial action coding system (FACS)^13^), are used to synthesize the pain facial expressions. To complete the loop, the physician perceives the visual facial expression cues generated by the MorphFace and adjusts their palpation behavior according to their internal model of what the facial expressions represent given the context. The internal model of the physician is typically based on factors such as hypothesis-driven exploration, sensory-motor coordination, and musculoskeletal dynamics.

Physical examinations are often performed in-person. However, with the growing shortage of physicians^14^ and the considerable challenges of conducting in-person patient examination due to global issues such as pandemics^15–17^, the time cost involved in face-to-face examinations^18^, and inaccessibility to examine the patients due to issues such as armed conflicts^19^, the need for remote diagnosis of patients has gained much attention in recent times. Further, remote diagnosis and treatment reduces the burden on hospitals and the risk of cross-infection during pandemics^20^. Specifically, remote palpation can be achieved through teleoperation using a robotic palpation device^21,22^. Importantly, such a system may have high telepresence^23^, including tactile information^24,25^. Consequently, several high-fidelity haptic feedback^26^ devices have been developed for use in conjunction with remote surgical systems. One simple format is a stimulation device that can be placed directly on top of the fingertip or wrapped around the control joystick^27^ and delivers a local vibration or compression^28^. In contrast, the most elaborate devices morph 2D surfaces to deliver tactile feedback by changing the surface’s geometry^29–31^. Alternatively, tactile information can be visualized by directly showing the tactile data as a color-coded matrix on a computer screen^32–34^ or creating a virtual simulation of the palpated object^35–37^. However, even though facial expressions provide effective feedback during palpation^38,39^, no existing teleoperated robotic palpation approaches incorporate facial expression as a feedback modality to control a remote palpation robot. In fact, these studies highlight how important facial expressions are as an information channel in in-person palpation examinations. Thus, all existing human–robot interaction approaches for remote medical examinations have one critical missing element-facial expressions. Here, we aim to leverage the inherent power of facial expressions as communicative signals to enhance the utility and effectiveness of these remote technologies.

In this paper, we investigate a “face mediated human–robot interaction” approach to perform teleoperated palpation tasks. With the advancement of videoconferencing technologies, considerable attention has been dedicated to bringing video-mediated communications closer to the real-world experiences of face-to-face interactions. For example, comparison of video, avatar, and robot mediated communications^40^ show that physical embodiment enhances social telepresence thereby achieving a closely resembled sense of real-world face-to face interactions. Exploiting these findings, we use a hybrid morphable robotic face: MorphFace^12^ to synthesize facial expressions of a patient under palpation examination in a remote location. A conceptual overview of the face mediated approach for remote palpation tasks is illustrated in Fig. 1. Facial expressions rendered on the robopatient face inform the user (e.g., a physician or medical student) about the condition of the patient under examination so that they can adjust their palpation action and control the remote robodoctor. Our face mediated approach is intuitive given that physicians often look for facial cues, during abdominal palpation to diagnose various medical conditions, particularly facial expressions of pain. Users of the system are thus expected to be able to quickly and efficiently decode the information presented by the robopatient’s facial expressions and adjust their palpation actions accordingly. Instead of using video recordings of facial expressions produced by individuals, we used the MorphFace which provides experimental control over the visual feedback provided to participants without risks to actual patients/participants at the robodoctor site. Using MorphFace provides rigorous experimental control over when and how the feedback is delivered to the user which can then be used to precisely characterize the users’ response to the facial expressions with high reproducibility. This would be challenging to achieve with actual patients due to potential uncontrolled variability in the facial expressions across participants. Further, given the risks associated with actual patients and the scarcity of simulated human patients during training of remote palpation examinations, the robopatient with the MorphFace can provide a better platform for training purposes.

We asked 17 healthy participants (6 females, 11 males) aged between 20 and 31 years (mean age: $24 \pm 3.4$ years)—students with no prior experience in remote medical examination or teleoperations and no history of sensorimotor impairments—to estimate the location of a hard nodule embedded in the silicone phantom (which resembles an abdomen of a patient with a painful hard nodule/mass) of the robodoctor (i.e., remote site) by palpating the silicone phantom place on the robopatient site while viewing the facial expressions displayed by the MorphFace. To compare our face mediated approach with existing alternative visual feedback formats, participants conducted the same task with a visual tactile map as feedback in a separate counterbalanced block. To evaluate the effectiveness of the face mediated approach, we measured five user behaviors: (1) the localization error of estimating the hard nodule positions, (2) accuracy of localizing the hard nodule, (3) nodule position estimation time, (4) number of unsuccessful trials, and (5) average proximity from the actual nodule position, and compared these across two feedback modes used during a teleoperated palpation task: facial expressions of pain displayed in the face mediated approach, and raw tactile sensor data displayed as a visual tactile map. When presented with the face mediated approach, most participants ($n=11/17$) showed lower median localization errors of estimating the hard nodule positions and significantly ($t_{16} = 2.6541, p = 0.0173$) fewer errors in localizing the hard nodule positions compared to when using the visual tactile map feedback. Moreover, most participants ($n=10/17$) estimated the hard nodule more accurately when using the face mediated approach as demonstrated by statistically significant higher accuracy ($t_{16} = 2.6565, p = 0.0172$) . Examination of nodule position estimation time and the number of unsuccessful trials did not reveal any significant differences between two feedback methods with $p>0.05$. Importantly, while the face mediated approach did not significantly shorten the exploration time (i.e., nodule estimation time), more accurate localization did not come at the expense of slower estimation time, thus demonstrating the advantage of face mediated approach in remote medical examinations. These results demonstrate the inherent power of facial expressions as communicative signals for visual feedback method to enhance the utility and effectiveness of human–robot interaction in remote medical examinations.

## Results

**Figure 2:**
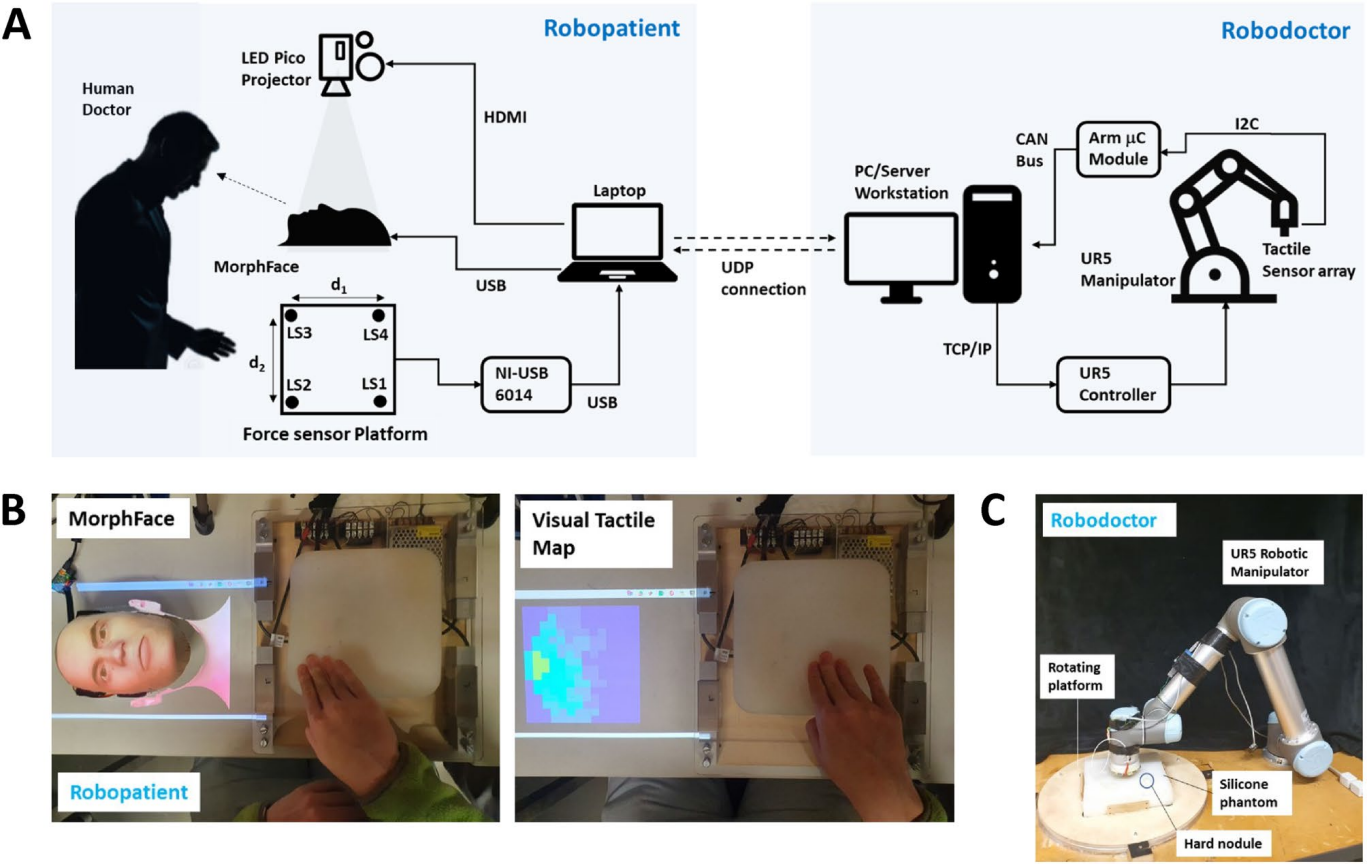
Overall system diagram and how the participants saw the two types of feedback during the experiment. **(A)** complete system diagram of the robopatient–robodoctor platform for remote palpation **(B)** Left shows how a participant sees the robopatient face during face mediated approach experiment. **(B)** Right shows How the visual tactile map would look like during the experiment. **(C)** The robodoctor site consists of a UR5 robotic manipulator, a rotating platform, and a silicone phantom with an embedded hard nodule.

Overall system diagram of the face mediated human–robot interaction for remote palpation is shown in Fig.  2A. The system consists of a robopatient where the user can perform palpation actions on a silicone phantom, and a robodoctor that mimics the palpation actions of the user based on the palpation force and position data acquired by a force sensor platform that sends this information to the robopatient via User Datagram Protocol (UDP). The robodoctor is a UR5 robotic manipulator with a tactile sensor array which comprises of 60 taxels on the end-effector which is used to measure the tactile pressure value when the user palpates on another silicone phantom and acquires pressure data that is then sent to the robopatient through the UDP. The received tactile sensor data is then used to map (encode) the information into a facial expression synthesized using the MorphFace—a hybrid morphable robotic face that we built in our recent work^12^.

**Figure 3:**
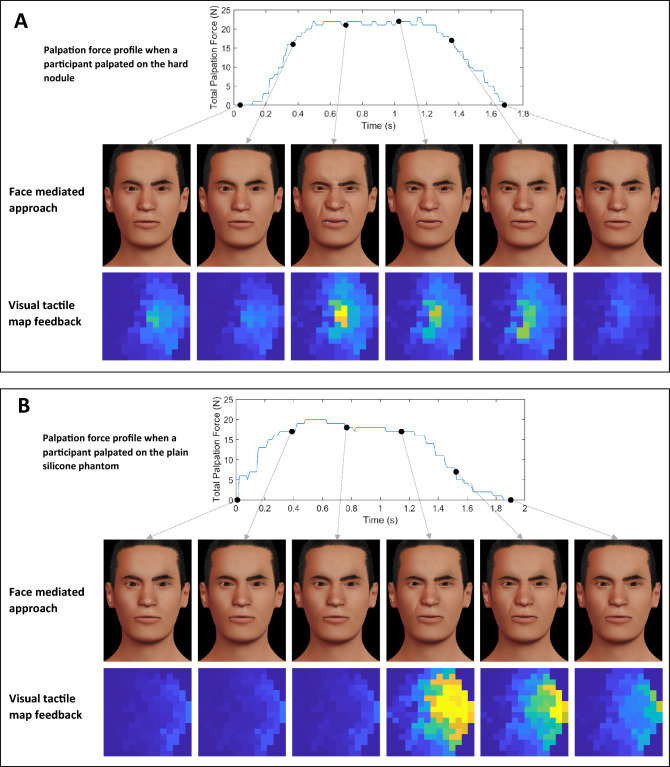
How pain facial expressions of the MorphFace in face mediated approach and corresponding visual tactile map feedback varies for different palpation forces. **(A)** top plot shows the palpation force profile when a participant palpated on the hard nodule and corresponding pain expressions rendered on the Morphface and respective tactile information displayed as the visual tactile map. **(B)** depicts the same information as in **(A)** but when a participant palpated on the plain silicon phantom. MATLAB 2020a (https://www.mathworks.com) is used to generate the images.

To test our approach, we conducted a user study with $n=17$ participants. In a teleoperated palpation task, participants estimated the location of a hard nodule embedded in the silicone phantom that was placed under the robodoctor (remote site) by palpating on the silicone phantom of the robopatient while viewing the facial expressions rendered by the MorphFace. Fig. 2B (Left) depicts how the participants saw the MorphFace during the experiment. To compare the effectiveness of the face mediated approach, participants completed the same task using a visual tactile map (Fig. 2B Right) as feedback instead of the face mediated approach. The visual tactile map is the direct representation of the data from the tactile sensor array. Specifically, it visualizes the sensor data without any post processing and thus provides the user with both force (tactile pressure data) and spatial information. Similar color map formats have been used in previous studies in palpation and/or medical examinations studies^32–34,41,42^. The visual tactile map does provide the user with how hard they pressed/palpated via color intensity, and where they pressed via pixels. Color intensity and the pixel patten in visual tactile maps thus inform the user about the stiffness distribution of the part of the phantom underneath the tactile sensor array. To demonstrate the difference between two feedback methods, Fig. 3A shows a sample palpation force profile when a participant palpated on the hard nodule and corresponding pain expressions rendered on the Morphface and respective tactile information displayed as the visual tactile map. Figure 3B depicts the same information when the participant palpated on the plain silicone phantom. As shown in Fig. 3B, when the tactile sensor array is pressed against plain silicone section, all tactile sensors get pressed and hence most pixels turn to yellow given the level of force. On the other hand, when the sensor array is pressed against an area where a hard nodule is embedded, high tactile values will register on the sensors over the nodule compared to sensors over the plain silicone sections. This directly reflects in the visual tactile map by a smaller area with pixels turned to yellow as in the Fig. 3A. The pattern of the colored pixels informs about the size/shape of the nodule as well as where the hard nodule is pressed against the tactile sensor array (e.g., whether the nodule is pressed over the centre of the tactile sensor array or near the perimeter of the sensor array). This allows the user to vary the palpation positions to approach towards the hard nodule. In the experiment, we asked participants to start palpating around the center of the phantom and use any exploration strategy to estimate the respective nodule position using two feedback methods. Each participant performed 20 trials with face mediated approach and 20 trials with visual tactile map as feedback with maximum trial duration set to 90 s (i.e., we instructed participants that they have 90 s to estimate the nodule otherwise the trials are considered as unsuccessful). We counterbalanced the order of the feedback method across participants to avoid any order effects.

A snapshot of the robodoctor site is shown in Fig. 2C. We fabricated one silicone phantom with a hard nodule (a 3D printed ABS ball with a diameter of 15 mm) embedded and placed on a rotating platform under the robodoctor. We manually rotated and locked the platform in one of the four orientations before each trial to change the position of the hard nodule across the experiment. We randomized the orientation of the platform for each trial across the experiment. A video taken during a trial with face mediated approach and visual tactile map feedback is presented in Movie S1.

**Figure 4:**
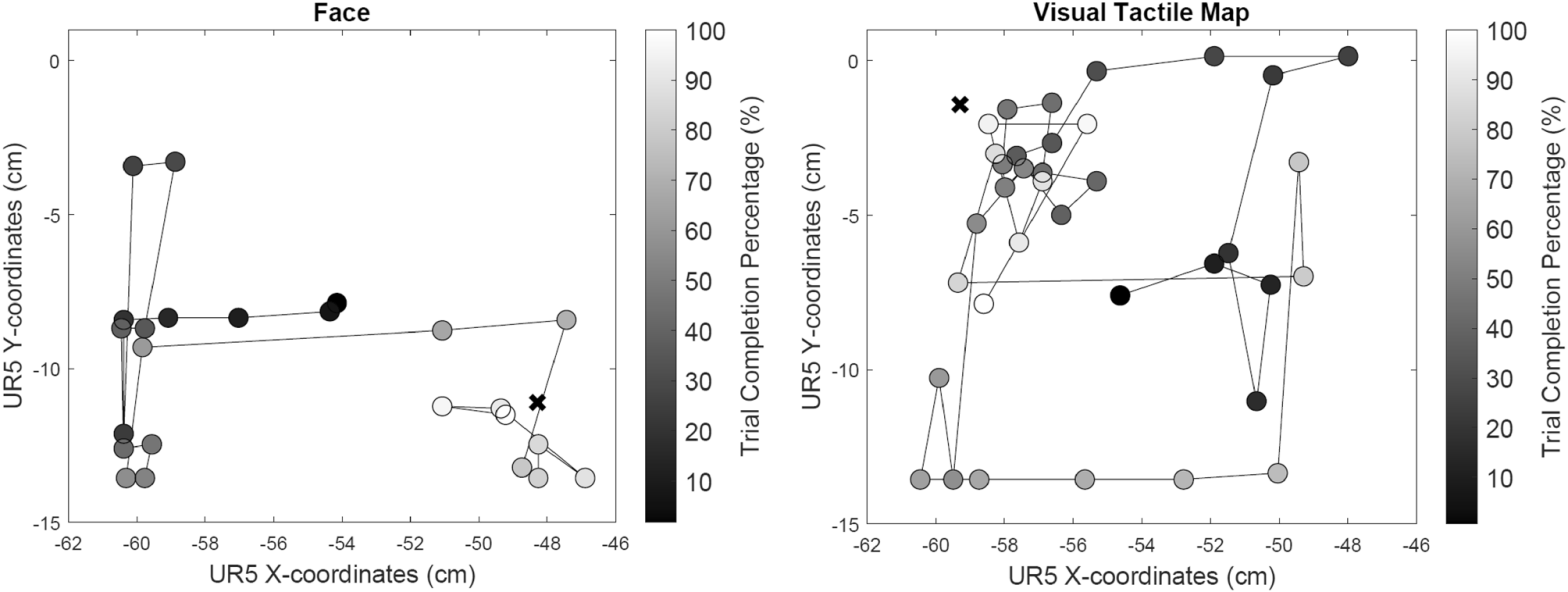
Exploration strategy used by one participant to estimate the location of a hard nodule in two feedback conditions. Left: when presented with face mediated approach; Right: when presented with visual tactile map feedback. Actual location of the hard nodule in respective trials is marked as a cross. Each filled scatter circle represents a palpation point and lines show the palpation path from start to end. Palpation points are color-coded according to the trial completion percentage (see color bar on right). MATLAB 2020a (https://www.mathworks.com) is used to generate the plots.

Figure 4 shows how a participant explored and estimated the hard nodule positions during a trial with the face mediated approach (Fig. 4 Left) and a trial with the visual tactile map feedback (Fig. 4 Right). The participant started palpation exploration from around the center of the phantom as instructed and was free to use any exploration strategy to find the hard nodule. Actual location of the hard nodule on respective trials are marked as crosses in the plot. Each filled scatter circle represents a palpation point and lines show the palpation path from start to end. In both trials, the participant estimated the position of the hard nodule within 90 s. Each palpation point is color-coded according to the trial completion percentage where lighter colors represents a higher percentage of trial completions. Results from these two trials show that the participant localizing the hard nodule position more accurately (based on the distance between the actual nodule position and the estimated nodule position) with face mediated approach compared to visual tactile map approach.

**Figure 5:**
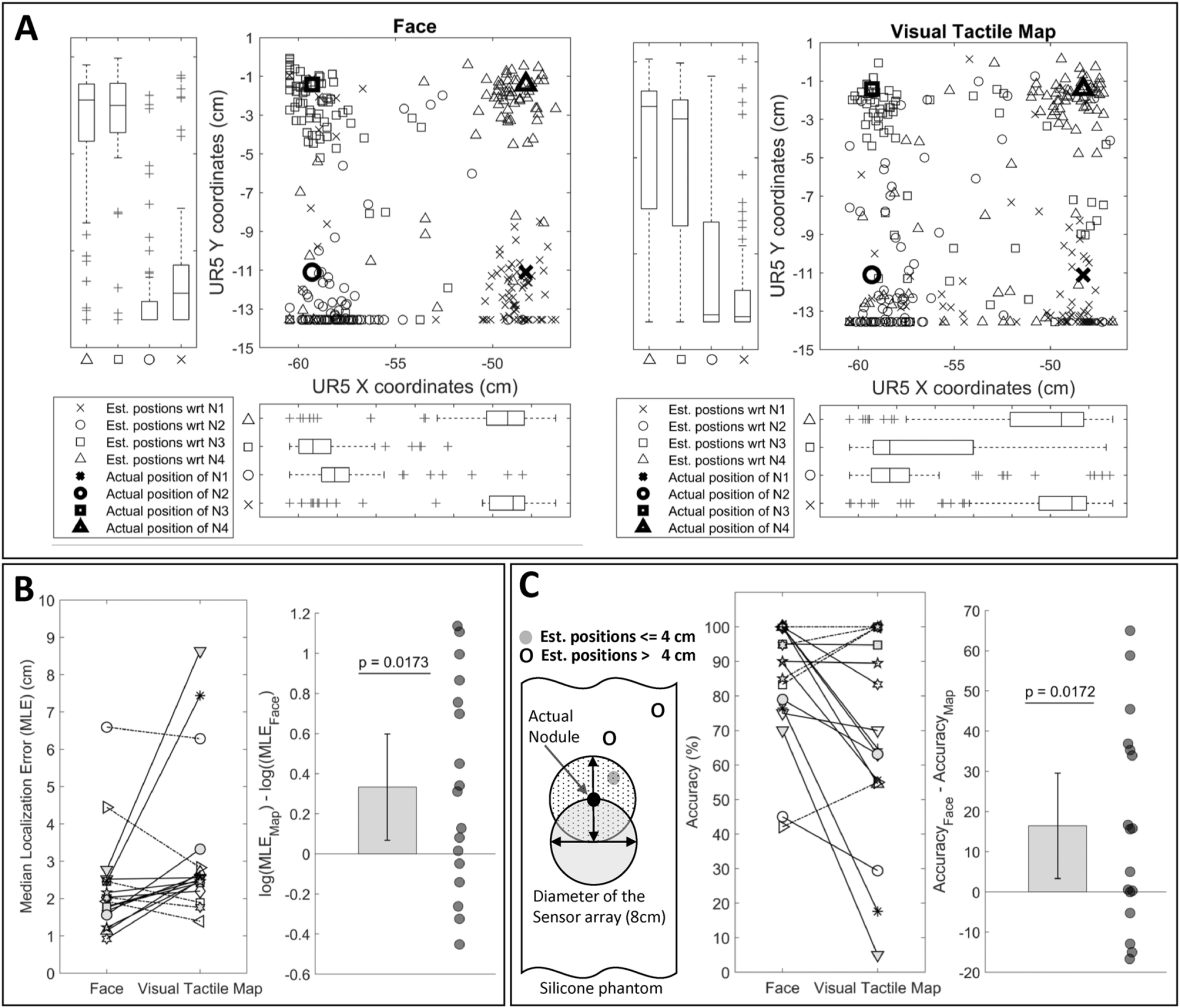
Estimated nodule positions, localization error and accuracy results for all participants **(A)** Estimated nodule positions by all participants during all successful trials (i.e., trials where the participant estimated the nodule position before 90 s) for each experiment. Box plots show the distributions of estimated nodule positions relative to actual nodule positions. **(B)** Left shows the paired plot for distribution of median localization error of successful trials of all participants. Each symbol represents an individual participant. **(B)** Right shows the log transformed distribution of the differences between the median localization error together with all data presented as a scatter plot. Thin black line represents the 95-confidence interval of the estimated mean difference. The p-value of the one-sample t-test ($t_{16} = 2.6541, p = 0.0173$) is shown above the plot. **(C)** Left we classified estimated nodule positions as accurate if they fell within the radius of 4 cm (as illustrates in the sketch) given that the diameter of the tactile sensor array was 8*cm*. Based on this notion. **(C)** Center shows the paired plot of accuracy for all participants between the two feedback methods. Each symbol represents an individual participant. **(C)** Right shows the difference of accuracy between face mediated approach and visual tactile map feedback together with all data presented as a scatter plot. Thin black line represents the 95-confidence interval. The p-value of the one-sample t-test ($t_{16} = 2.6565, p = 0.0172$) is shown above the plot. MATLAB 2020a (https://www.mathworks.com) is used to generate the plots.

Figure 5A shows the estimated nodule positions across all participants during all successful trials for each feedback format (total number of successful trials for face mediated approach and tactile feedback method = 288 and 307, respectively). On each trial, we presented one hard nodule in the phantom placed under the robodoctor-the location of this nodule is represented as a bold symbol in the scatter plots (see legend to bottom left). Therefore, each of bold symbols indicate the actual nodule positions during all successful trials. The estimated nodule positions of the participants are shown as symbols. Symbol type represents the respective nodule (i.e., type of bold symbols). Box plots show the distributions of respective estimated nodule locations relative to UR5 Y coordinates and X coordinates, respectively. We observed higher variance in the estimated positions when using the visual tactile map feedback compared to the face mediated approach.

To measure the participants’ accuracy in locating the hard nodule, we measured the distance between the actual nodule position and the estimated nodule position across all trials and examined the localization error of estimating the hard nodule for each participant. Figure 5B left compares the median localization error of two feedback methods for all participants. Solid lines show the participants with fewer median localization errors when using the face mediated approach compared to the visual tactile map feedback ($n = 11/17, Hedges'g = [0.04, 0.97]$); dashed lines show the participants with the opposite pattern ($n = 6/17$). Therefore, most participants localized the targets more accurately with face medicated approach compared to the visual tactile map feedback. We also analysed whether there was any order effect on the results. As shown in Fig. S1 (in Supplementary Information), we did not observe any order effect. A one sample t-test applied to the log transformed distribution of the differences between the medians of the two feedback formats (Fig. 5B right) showed a statistically significantly positive difference across participants ($t_{16} = 2.6541, p = 0.0173$).

The tactile sensor array used has an approximate diameter of 8 cm. Given that any estimation within this region could be caused by the actual nodules, we considered any estimated nodule position within a 4 cm radius circle from the actual nodule position (see Fig. 5C Left) as an accurate estimation. Thus, we calculated the accuracy of estimation as the ratio between estimated trials falling within the 4 cm circular radius area and all successful trials. Figure 5C Center shows the paired plot with the accuracy of two feedback methods connected for all participants. Most participants ($n = 10/17$) showed higher accuracy when using the face mediated approach, as demonstrated by performance that is greater than or equal to the visual tactile map feedback by at least 5%. A one sample t-test applied to the differences of accuracy between the two feedback methods (Fig. 5C Right) showed statistically significant higher accuracy ($t_{16} = 2.6565, p = 0.0172$) when using the face mediated approach (mean accuracy 83.8%) compared to the visual tactile map feedback format (mean accuracy 67.4%).

**Figure 6:**
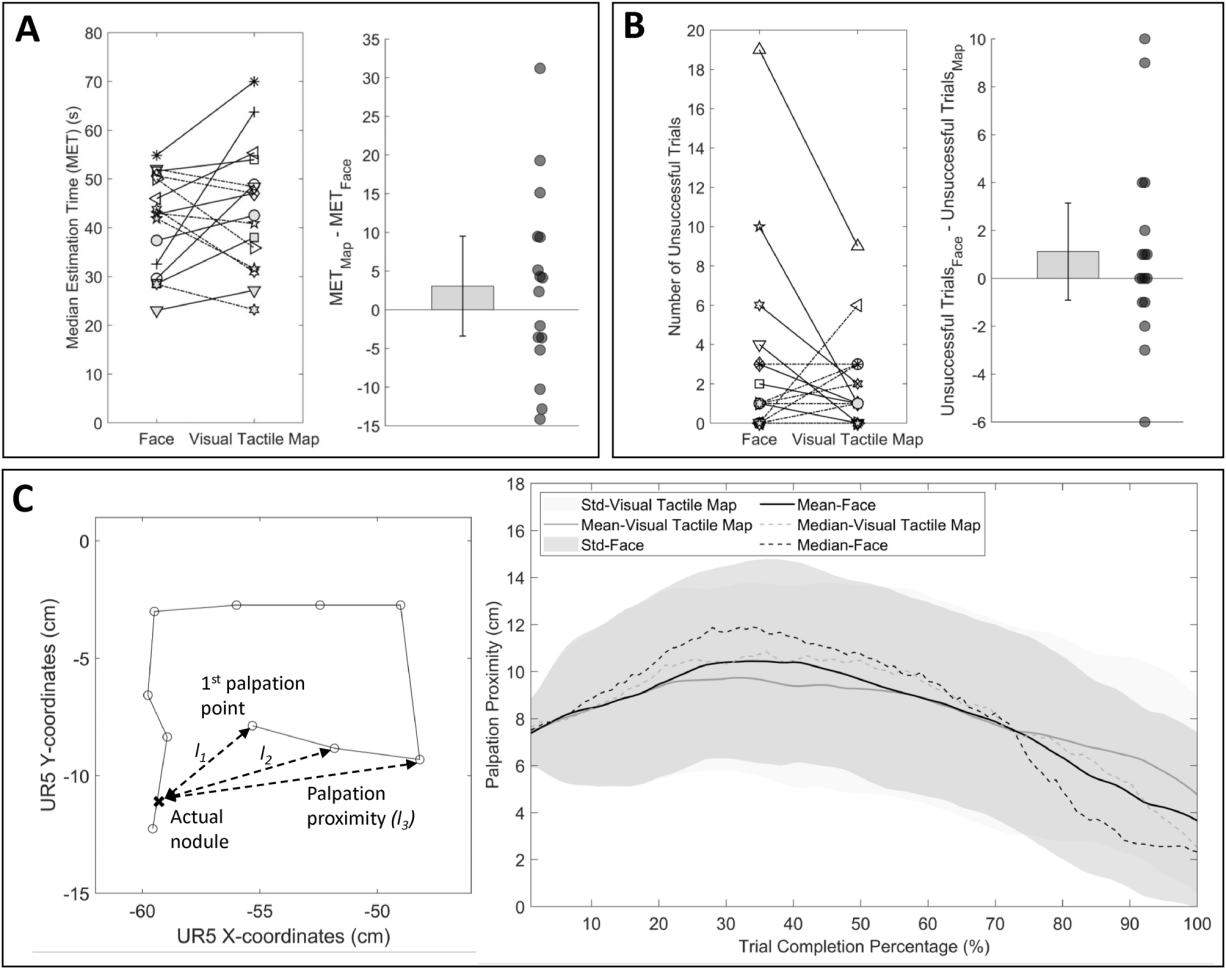
Results of nodule estimation time, number of unsuccessful trials and palpation proximity for all participants. **(A)** Left shows the paired plot of the distribution of median estimation time of successful trials of all participants. Each symbol represents an individual participant. **(A)** Right shows the distribution of the differences between medians of estimation time together with all data presented as a scatter plot. Thin black line represents the 95-confidence interval. **(B)** Left shows the paired plot of the distribution of the number of unsuccessful trials of all participants. Each symbol represents an individual participant. **(B)** Right shows the distribution of the differences between the number of unsuccessful trials of two feedback formats as a scatter plot. The thin black line represents the 95-confidence interval. **(C)** Left illustrates an example recorded palpation exploration profile from one trial performed by a participant. We defined palpation proximity as the length between current palpation point and the actual nodule position on a given trial. **(C)** Right average proximity from the actual nodule position is plotted against the proportion of trials completed. MATLAB 2020a (https://www.mathworks.com) is used to generate the plots.

In medical diagnosis, both accuracy and the time to arrive at a decision is important. A more accurate but slower approach may not be as effective as a less accurate but faster approach. Therefore, we compared the two feedback methods in terms of nodule position estimation time by calculating the duration between the participant starting their first palpation and final palpation before making their decision as to the location of the nodule. On most trials, participants started their palpation around center region as instructed (median palpation starting position across all successful trials for all participants in both the feedback methods was $X =-\,54 \,\text{cm}$ and $Y =-\,8\, \text{cm}$). While starting palpation position do not influence localization error or accuracy, it could influence nodule estimation time and palpation strategy. Thus, to have a meaningful comparison between two feedback methods, we excluded any trials where the participant started outside the area defined by $X = -\,54 \pm 2.5\, \text{cm}$ and $Y = -\,8 \pm 2.5\, \text{cm}$ from the analysis. Consequently, we removed one participant from the analysis, resulting in a total of 258 and 272 trials for analysis with the face mediated approach and visual tactile map feedback, respectively (exclusion trial percentages for face mediated approach and visual tactile map feedback were 10.4% and 11.4% respectively).

Figure 6A Left shows the comparison of individual median estimation time across all trials for all the participants between two feedback approaches. Half of the participants ($n = 8/16$) localized the target faster with the face mediated approach compared to the visual tactile map feedback (i.e., showed positive effects between visual tactile map feedback and face mediated approach, $Hedges' g = [0.29, 1.52]$). However, the advantage was not statistically significant (one sample t-test: $t_{15} = 1.0040,p = 0.3313$ ) (Fig. 6A Right). On average, participants completed all trials in 42*s* when using face mediated approach and 43 s when using the visual tactile map feedback. Importantly, even though the advantage of the face mediated approach in terms of shortening the exploration time was not statistically significant, the fact that the reduced localization error did not come at the expense of slower estimation provides an additional support for the advantage of face mediated approach.

Furthermore, the extent to which participants could estimate the nodule positions within the designated 90 s-time frame could provide insights into the complexity of the two feedback methods. Therefore, we examined the number of unsuccessful trials across the two feedback methods. Figure 6B Left shows the number of unsuccessful trials for each feedback format for each participant separately. A one-sampled t-test showed no significant differences ($t_{16} = 1.1663, p = 0.2606$) in the number of unsuccessful trials across feedback formats (Fig. 6B Right). We observed an average of 3 unsuccessful trials out of twenty trials across participants for the face mediated approach and 2 unsuccessful trials for the visual tactile map feedback.

Figure 6C Left shows an example recorded palpation exploration profile from one trial performed by a participant. To characterize how the two feedback methods affected the exploration strategies of the participants, we calculated the distance of each palpation point from the actual nodule position in the trial to represent palpation proximity (e.g., $l_{1},l_{2}, l_{3}$ and so on). We calculated the proximity of the participants’ palpation to the actual nodule position over the course of each trial to thus characterize the participants’ exploration strategies according to the two feedback methods. Figure 6C Right shows the palpation proximity of each palpation from the actual nodule positions as a function of the percentage of trials completed. We used all trials that we considered for characterizing the nodule estimation time in this analysis. For both feedback approaches, participants tended to explore areas away from the actual nodule position until 30–40% of the trial time. Towards conclusion of the trial, participants palpated more toward the actual nodule as they refined their estimation of the nodule position based on the feedback method. Participants tended to approach the estimated nodule position more quickly with the face mediated approach compared to the visual tactile feedback, in the second half of the trial time.

## Discussion

Abdominal palpation is one of the primary physical examination methods used by physicians to examine the condition of the abdomen. Viewing the patient’s facial expressions during this manual palpation provides a critical source of feedback to assess a range of medical hypotheses for diagnosis. Given the growing shortage of physicians^14^, the chronic issues such as pandemics^15–17^, cost and time cost involved in face-to-face examinations^18^, inaccessibility to examine the patients due to issues such as armed conflicts^19^, the need for remote diagnosis of patients has gained much attention in recent times. Remote palpation examination is one such approach that is growing in interest for remote diagnosis. Though different solutions have been proposed to develop remote and/or robotic palpation devices, none have considered incorporating facial expression as an effective and intuitive feedback modality. In this work, we do exactly that and present an approach called “face mediated Human–robot interaction” for remote palpation tasks.

The face mediated approach relies on two main sub systems: robopatient and robodoctor. The user can palpate on the abdominal phantom of the robopatient and observe the facial expressions synthesize by the Morphface. This provides the user with an intuitive system that resembles the scenario of in-person palpation on a human patient and could engender interpersonal trust via physical embodiment and control of the system in mediated communication^43^. Further, such teleoperated robots with a realistic human appearance can improve social telepresence compared to audio or video conferencing^44^. These points highlight the importance of having a physically embodied robopatient for teleoperated palpation.

In the experiments, participants estimated the position of a hard nodule embedded in a silicone phantom using one of two visual feedback methods in two separate tasks: facial expressions rendered via the MorphFace in the face mediated approach vs visual tactile map. Results showed that participants localized the hard nodule significantly more accurately when using the face mediated approach compared to the visual tactile map. Participants formed two groups based on their localization error with the two feedback methods. Most participants performed better in finding the nodule positions when using the face mediated approach with around half ($n = 9/17$) reporting in post-experiment feedback that they found the face mediated approach to be a more intuitive format than the visual tactile map.

In contrast, the remaining participants localized the hard nodules more accurately when using the visual tactile map feedback. Note that the visual tactile map represents all tactile data including the spatial information and force values of the palpation. Thus, representing tactile sensor data as a visual map has its own merits because it can inform the user about how to vary their palpation positions to estimate the hard nodule. However, the disadvantages are that the user must simultaneously consider several pieces of information including the color, position, and overall changes of pixel patterns to estimate nodule position. Given that the tactile sensor array used here consists of 60 taxels and that we encoded the tactile data from the sensors into a facial expression of pain synthesized using four pain-related AUs, this process can be considered a data compression. We speculate that this data compression leads for better understanding of tactile data by the participants.

Though localization error differed significantly across feedback methods, we found no statistically significant differences in nodule position estimation time, showing that the examination does not take longer with the face mediated approach compared to the visual tactile map feedback. This is important by itself from the clinical perspective because some physicians prefer a faster than more a precise method. While the speed-accuracy trade-off, as formally manifested in Fitts’ Law^45,46^, is generally observed for motor rather than perceptual tasks, it could play a role here given that palpations comprise both motor and perceptual tasks. If so, the fact that the participants perform better with the face mediated approach without being slower suggests that they are overall more skillful in using the face mediated approach.

How participants look for information to inform their decisions could be based on their palpation strategy. The participants started their palpation actions from the center area of the phantom as instructed at the beginning of the experiment. For both the feedback approaches, the participants generally explored areas away from the actual nodule positions until 30–40% of the trial time. As the trial concluded, participants began to palpate toward the actual nodule as they refined their estimation of the nodule position. Participants also tended to approach the estimated nodule position more quickly with the face mediated approach compared to the visual tactile map feedback during latter half of the trial. This result suggests that participants used a less cautious exploration strategy when using the visual tactile map feedback and a more direct strategy when using the face mediated approach.

In this work, we showed that even with the most primitive use of facial expressions as presented in the paper, there are significant differences in performances of participants from the visual tactile map which is another most primitive display. The facial pain expressions as in this work are synthesized based on a set of AUs which have been identified as relevant to pain^47^ are based on the taxonomic Facial Action Coding System (FACS)^13^. These pain facial expressions are then displayed on a specific face identity, thus comprising a visually complex stimulus, providing a broad range of information to an observer^48^. Though human facial expressions are complex signals, as described here, humans have a highly developed ability to extract information from them efficiently and effectively^49,50^. This evolved expertise with faces in particular may suggest the intuitiveness with facial displays especially given that palpation examinations often involve observing facial reactions as feedback. However, it is possible that the face mediated approach performed better because it presented a unidimensional variable of pain intensity rather than several variables as in the visual tactile map. Future studies are needed to ascertain between these two interpretations. The comparison of two feedback approaches may not the ideal assessment to claim that the face mediated approach is the optimal choice as a display of the palpation feedback, but there are many additional investigations necessary with higher granularity of facial expressions and visual tactile map parameters, in order to find out exact causes of where the intuitiveness of the MorphFace comes from. Even though we have not yet found the optimal MorphFace to be used in the face mediated approach, this article provides a significant insight into the importance of intuitive display and the method of assessment, which should be an initial significant cornerstone of the future research.

Despite these promising findings, some limitations invite caution. Our main goal was to compare palpation behavior and nodule location accuracy when using the face mediated approach and the visual tactile map feedback. However, as highlighted in the introduction, palpation relies on a combination of different feedback sources, including visual feedback via facial expressions, haptic and auditory feedback. In the current work, we focused specifically on visual feedback to evaluate the performance of the face mediated approach compared to an alternative visual feedback method. Future work will compare the face mediated approach with a haptic feedback system, and examine how the face mediated approach performs together with a haptic feedback system during remote medical examination. As an initial test, we synthesized one face identity (a white male) even though the Morphface can render many other face identities of different ethnicities in the face mediated approach. Recent studies^51,52^ have reported the variability of interpretation of pain facial expressions. Thus, face identity feature variations (e.g., sex, ethnicity, age) in the MophFace could provide the participant/observer with more information about pain experience and this will be examined in future work. We also did not consider whether and how participant demographics might influence performance when using the different feedback methods and interact with the face identity used. We will address this issue in future work by including a more diverse range of face identities and participants. Another limitation is that the participants had no prior experience in palpation examination. Participants were students, mainly from an engineering background with no prior experience in remote medical examination and teleoperations. Given their exposure to different data visualization techniques such as matrices and color maps, it is likely that they had a good understanding of how to interpret the color maps. In contrast, healthcare professionals who have extensive training in interpreting facial expressions during medical examinations could perform better with the face mediated approach compared to visual tactile map. Future work that evaluates the proposed approach with senior medical students and/or physicians who have undergone training in palpation would provide more insights into the utility of this feedback format on palpation strategy and performance. Nevertheless, we demonstrate the overall feasibility and effectiveness of face mediated human–robot interaction approach for remote medical examinations.

## Methods

### Participants

Seventeen healthy participants (6 females, 11 males) aged between of 20 and 31 years (mean age: $24 \pm 3.4$ years)—students with no prior experience in remote medical examination or teleoperations and no history of sensorimotor impairments took part in the experiment. To reduce the risks associated with the spread of the coronavirus (COVID-19) we limited the number of participants enrolled. Sixteen participants self-reported right-hand dominance. All the participants had normal or corrected to normal vision. We provided all participants with instructions about the experiment procedure in both written and oral format. All participants provided written informed consents. The experiment protocol was approved by the ethics committee of Department of Engineering, University of Cambridge, UK. All experiment protocol were performed in accordance with the relevant guidelines and regulations.

### Experimental set-up

Overall system as a block diagram is shown in Fig. 2A. It consists of two main systems: a robopatient and a robodoctor. The two systems are connected each other using an UDP connection realized with a mobile Wi-Fi. The robopatient is implemented in the MATLAB 2020a (*MathWorks Inc.*) environment whereas for the robodoctor a custom written python program is used. Following subsections detail on the subsystems of the set-up.

### Robopatient

The robopatient consists of a hybrid morphable robotic face: MorphFace to synthesis facial expressions, a force sensor platform, and a silicone phantom to replicate the patient’s abdomen. An overview of the total system is shown in Fig. S2 (in Supplementary Information). The force sensor platform is developed to measure the palpation force and positions during user palpation. The force sensor platform is made with four single axis Tedea Huntleigh 1040 (20 kg) load cells mounted on the four corners of a wooden platform where distances between two adjacent load cells are 290 mm and 310 mm. A National Instrument DAQ (USB-6341) with MATLAB 2020a is used to acquire the signals from the load cells at a sampling rate of 1000*Hz*. The force values from the load cells are used to calculate the total palpation force ($F_{tp}$) and palpation position ($x_p$ and $y_p$) as below. Here, to minimize the noise on these calculations, any load cell values when total palpation force is less than 5*N* have been neglected based on the observations from pilot experiments.

1 $$\begin{aligned} F_{tp}&= \sum ^4_{j=1}F_j, \end{aligned}$$

2 $$\begin{aligned} x_{p}&= (F_2+F_3)\frac{d_1}{F_{tp}}, \end{aligned}$$

3 $$\begin{aligned} y_{p}&= (F_3 + F_4)\frac{d_2}{F_{tp}}, \end{aligned}$$

where $F_j$ is force readings of *j*th load cell mounted at the four corners of the force sensor platform, and $d_1$, $d_2$ are the distance between two adjacent load cells. A square shape ($205 \times 205 \times 30$ mm) silicone block fabricated using Ecoflex 00-20 (*Smooth-On, Inc.*) is placed on the load cell platform and used as the abdominal phantom of the robopatient. Then, we re-scaled the range of the palpation positions so that when user palpate on the four corners of the phantom of the robopatient, robotdoctor does the palpation motion on the respective four corners of the phantom placed on the robodoctor site. Finally, the total palpation force ($F_{tp}$) and re-scaled palpation positions ($x_{rp}$, $y_{rp}$) are sent to the robodoctor via the UDP connection.

MorphFace is a controllable 3D physical-virtual hybrid face that can represent facial expressions of pain displayed on six different ethnicity-gender face identities: female and male faces of White, Black, and Asian ethnicity. The pain facial expressions are realized by 4 facial action units: AU4 (Brow Lowerer, AU7 (Lid Tightener),AU9 (Nose Wrinkler) and AU10 (Upper Lip Raiser) with the pain intensity (*PI*) ranging from 0 (no pain) to 100 (maximum pain)^53^. More details of MorphFace can be found in our recent work^12^. We included only the white male face identity for this study.

We used the tactile sensor data received via UDP from the robodoctor to map the facial expression intensity of the MorphFace. First, we re-arranged the tactile data received in terms of 60 taxels ($S_j$) (see more information in the Robodoctor section) into a 16-by-14 matrix *V* to match with the shape of the overall physical sensor array and the arrangement of each individual sensor mounted within the physical sensor array. When the sensor array is pressed against a uniform surface, all taxel values should be approximately equal. On the other hand, if it is pressed against a surface with uneven stiffness, the taxels over the hard area should register higher values compared to taxels on the remaining area. The location of the hard area/point while the sensor array is pressed can be calculated using the distance between the maximum taxel location (or index) and the center taxel location within the matrix *V* . Given this, we mapped the facial expression pain intensity (*PI*) and taxel data using following equations.

4 $$\begin{aligned} PI&= \beta ~PI_{filtered}, \end{aligned}$$

5 $$\begin{aligned} P_{raw}&= [(Max(V) - Mean(V)](1-d_{max}), \end{aligned}$$

6 $$\begin{aligned} d_{max}&= \frac{\sqrt{(V_{i,max}-V_{i,center})^2 + (V_{j,max}-V_{j,center})^2}}{d_{n}}, \end{aligned}$$

where $\beta = 10$ is the scaling factor to map the range of between 0 to 100, $PI_{filtered}$ is the filtered (using a moving average filter of window size 10) output of $PI_{raw}$. The *Max*(*V*) is the maximum taxel value, *Mean*(*v*) is the mean of the taxel values, $d_{max}$ is the normalized (between 0 to 1) distance between the location of maximum taxel and the location of the center taxel ($V_{i,center}, V_{j,center}$) within *V*. The ($V_{i,max}, V_{j,max}$) is the position of the maximum taxel within *V* whereas $d_{n}$ denotes the distance between the center taxel to the furthest taxel within *V*.

### Robodoctor

The RobotDoctor consists of a 6 degrees of freedom (DoF) UR5 Robotic Arm (*Universal Robots*) that can perform complex end-effector trajectories, and a tactile capacitive sensor array. The robot is placed in front of the workbench used to perform the experiments and it is directly controlled by a server workstation via TCP/IP. The sensor used to collect haptic data is a hexagonal capacitive sensor array with 60 tactile elements, or ‘taxels’, providing high sensitivity and spatial distribution over the surface of the sensor. The sensor provides measurement with a resolution of 16 bits corresponding to a variation of capacitance proportional to the pressure acting on top of the sensor. Details of the specific sensor and its fabrication have been previously reported^54,55^.

The desired end effector positions $x_{d}$ and $y_{d}$ for the controller of UR5 are calculated using a simple linear transformation from the Robopatient coordinate system to the Robodoctor, as follows:

7 $$\begin{aligned} {\left\{ \begin{array}{ll} x_{d} &{}= -y_{rp}\\ y_{d} &{}= x_{rp} \end{array}\right. }, \end{aligned}$$

where $x_{rp}$ and $y_{rp}$ are the coordinate in the Robopatient coordinate systems, and $x_{d}$ and $y_{d}$ are the same coordinates mapped to Robodoctor.

On the other hand, $F_{tp}$ is mapped to its correspondent target taxels’ total value $Tx^T_{tot}$. During the initial calibration of the system we indent the silicone phantom of the desired maximum possible displacement $z_{max}$, selected a priori, and we compute the total sum of the taxels’ values in that position $Tx^{max}_{tot}$. During the trials, $F_{tp}$ is re-scaled into a $Tx^T_{tot}$, and then used for the position control, as follows:

8 $$\begin{aligned} Tx^{max}_{tot}&=\sum ^N_{j=1}Tx_j(z_{max}), \end{aligned}$$

9 $$\begin{aligned} Tx_{tot}&=\sum ^N_{j=1}Tx_j(z), \end{aligned}$$

10 $$\begin{aligned} Tx^T_{tot}&= \alpha Tx^{max}_{tot} \frac{F_{tp}}{F_{tp_{max}}}, \end{aligned}$$

11 $$\begin{aligned} L&= \sqrt{(x^T - x)^2 + (y^T - y)^2 }, \end{aligned}$$

12 $$\begin{aligned} z^T&={\left\{ \begin{array}{ll} z_0 - (Tx^T_{tot} - Tx_{tot}) &{} d< d_0 \wedge F> F_0\\ z_0 &{} d> d_0 \wedge F > F_0\\ z_0 + \Delta z_0 &{} F < F_0 \end{array},\right. } \end{aligned}$$

where *L* is the distance from the target point projected in the *xy* plane, $F_{tp_{max}}$ is the saturation value of the force registered by the RobotPatient and $F_0$ is the activation threshold; $x^T$, $y^T$, and $z^T$ are the target coordinate, whereas *x*, *y*, and *z* are the current ones; $d_0$ is the maximum radial distance for which the pressing motion is started, introduced to avoid sliding and friction between the end-effector and the phantom; $z_0$ is the *z* coordinate of the surface of the phantom $\Delta z_0$ is a safety margin fixed a priori and $\alpha$ is a scaling factor to rescale the range of motion of the robot. The collected data from the sensor array are normalized with respect to the maximum and minimum of each individual taxel and final 60 taxel data ($S_{j} ; j=1,2,\ldots ,60$) are sent to robopatient via UDP.

### Visual tactile map

The visual tactile map is the direct representation of the data from the tactile sensor array. The visual tactile map is generated using the matrix *V*. We use *imagesc* function (color limit: 0–10) in MATLAB 2020a to display the matrix *V* as a color map and projects it on to the table during experiments. We use the MATLAB’s default *parula* colormap array as the color scheme where it maps the lowest tactile sensor values to dark blue, then continuously increasing via green and the highest tactile sensor values map to bright yellow.

### Experimental procedures

The experiment consisted of two main tasks. In one task, participants palpated the abdominal phantom of the robopatient and estimated the location a hard nodule embedded in a silicone phantom place under the robodoctor based on the facial expressions feedback rendered on the MorphFace. In the other task, participants repeated the same task using a visual tactile map projected on to the table instead of the MorphFace. We counterbalanced the order of two tasks across participants to control for potential learning and order effects. We fabricated the silicone phantom ($215 \times 215 \times 35$ mm) placed under the robodoctor using Ecoflex 00-20 (*Smooth-On, Inc.*). The hard nodule comprised a 3D printed ball (ABS) (diameter = 15 mm). Each task comprised a total of 20 trials with a maximum duration of 90 s per trial (i.e., we instructed participants that must estimate the location of the nodule within 90 s otherwise the trial is considered as unsuccessful). At the start of each trial, we randomized the orientation of the phantom at the robodoctor site to one of 4 positions. A flow chart of the experimental sequence is depicted in Fig. S3 (in Supplementary Information). At the start of each session, we familiarized the participants with each feedback system by instructing them to palpate on an area with plain silicone and directly over the hard nodule and to observe the feedback variations and range in each format separately. Each trial started with a auditory cue (a beep) to indicate the start of the trial. Participants then started their palpation exploration from around the center of the phantom and were free to use any exploration strategy to find the hard nodule. Once the participant had decided that they had found the position of the hard nodule, they palpated that position once and informed the experimenter verbally. The experimenter then concluded the trial by pressing any of a laptop keyboard. If the participant did not find the position of the hard nodule within 90 s, we considered the trial as unsuccessful. We recorded all data received from the robopatient via UDP, robodoctor data including UR end effector positions, tactile sensor data, actual nodule positions and respective time stamps for each trial.

## Supplementary Information

Supplementary Information.

Supplementary Video S1.

## Data Availability

All the data needed to evaluate the study are in the main text and Supplementary Materials. The raw datasets and codes used to analyze data and to produce plots during this study are available in OSF repository: https://osf.io/6m9d7/.
