# Supplementary material for: Face mediated human–robot interaction for remote medical examination
Source: Sci Rep. 2022 Jul 22;12:12592. doi: 10.1038/s41598-022-16643-z (PMC9307637; doi:10.1038/s41598-022-16643-z)
Supplement: Supplementary file 1 — Supplementary Information. [file 41598_2022_16643_MOESM1_ESM.pdf]

Supplementary Information

S1 Supplementary Figures

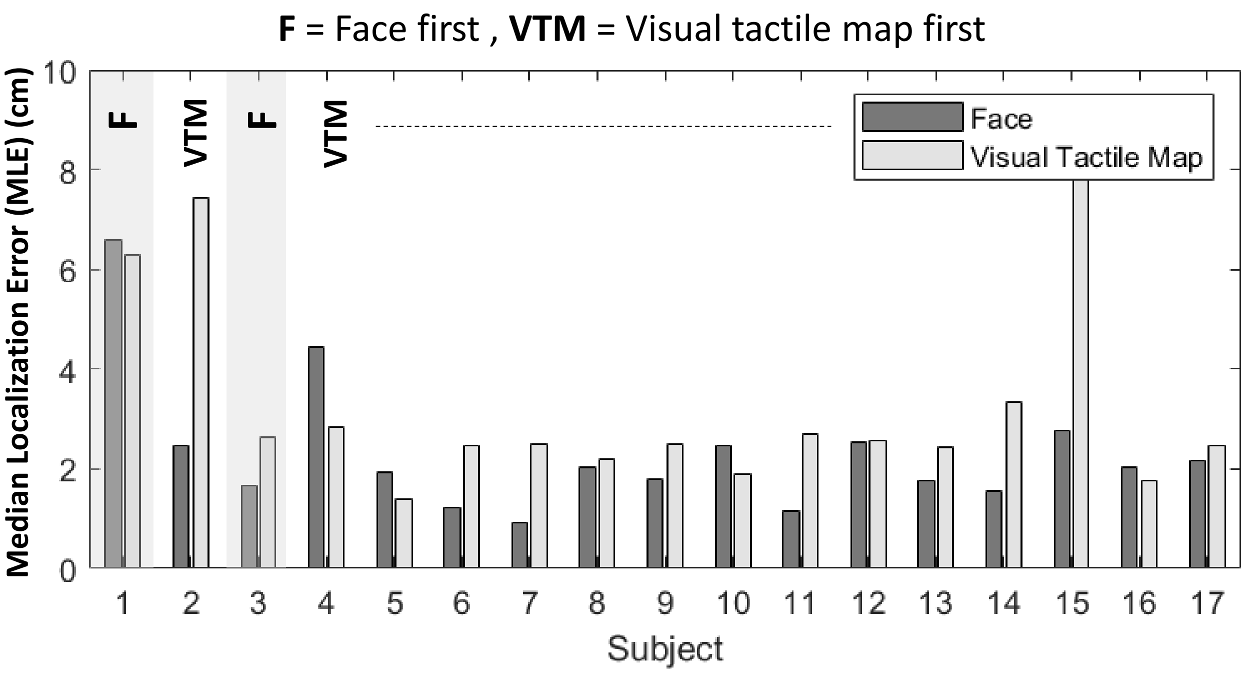

Figure S1. Median localization error during two feedback approaches for all the participants. Participants with odd subject numbers performed the face mediated approach first while participants with even subject numbers conducted the visual tactile map experiments at the start.

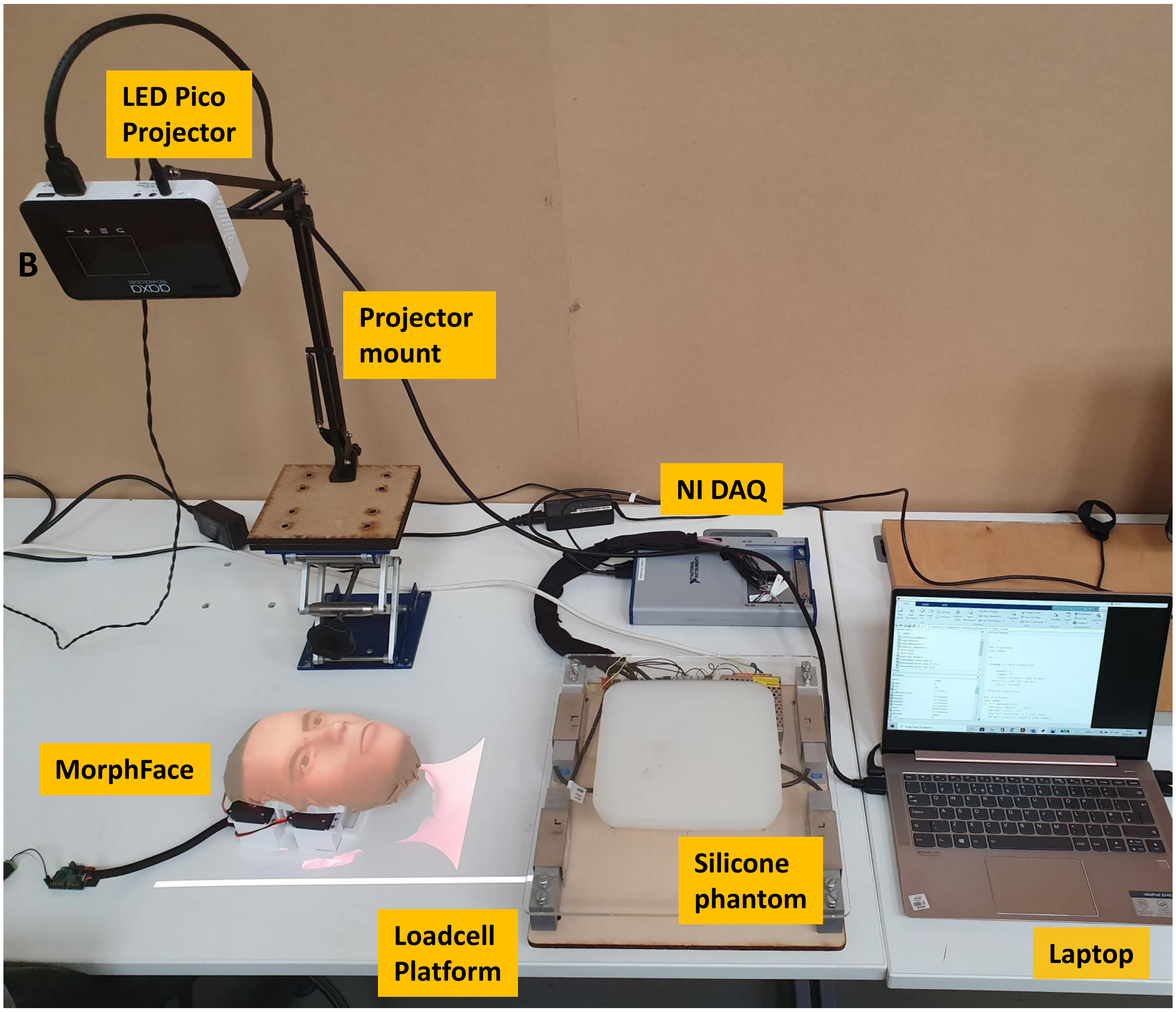

Figure S2. Overview of the robopatient hardware system. It mainly consists of a robotic face (MorphFace), a force sensor platform and a silicone phantom to simulate the patient's abdomen. The physical robotic face has four degrees of freedoms (eyes, nose, mouth, and chin) which are controlled via tendons connected to 4 mini RC servos (MG996R) to span $\sim$ 85% of facial variations across gender and ethnicity. The face can represent facial expressions of pain displayed on six different ethnicity-gender face identities: female and male faces of White, Black, and Asian ethnicity. The RC servo motors are driven by a Pololu Maestro 12-channel servo driver which is connected to the laptop. Details such as skin colour, skin texture, and facial expressions are synthesized by a virtual model projected onto the 3D physical face via a front-mounted LED projector (*AAXA P300 Neo Smart Android Mini Pico Projector, AAXA Technologies*) to obtain a hybrid controllable patient face. In the virtual model, pain facial expressions are realized by 4 facial action units (AUs): AU4 (Brow Lowerer, AU7 (Lid Tightener), AU9 (Nose Wrinkler) and AU10 (Upper Lip Raiser) with the pain intensity (PI) ranging from 0 (no pain) to 100 (maximum pain).

S2 Methods

Data Analysis

We analysed all data recorded during the experiments using MATLAB 2020a. First, we filtered the palpation force data using a moving average filter of sample window 20 (using *smooth* function). To detect the palpation peaks, we used the *findpeak* function in MATLAB with parameters set to 1, 2 and 50 for minimum peak prominence, minimum peak height, and minimum peak distance, respectively. Given that resulting detection contained multiple peaks at each palpation action, we implemented a custom algorithm to select the maximum peak within trials that registered a palpation force greater than $5N$. Based on the final detected peaks, we calculated the indexes and used them to find the respective time stamps in seconds and x, y positions of the robot manipulator. Finally, we saved the palpation force peaks, time stamps for peaks, respective x, y positions of the robot, and

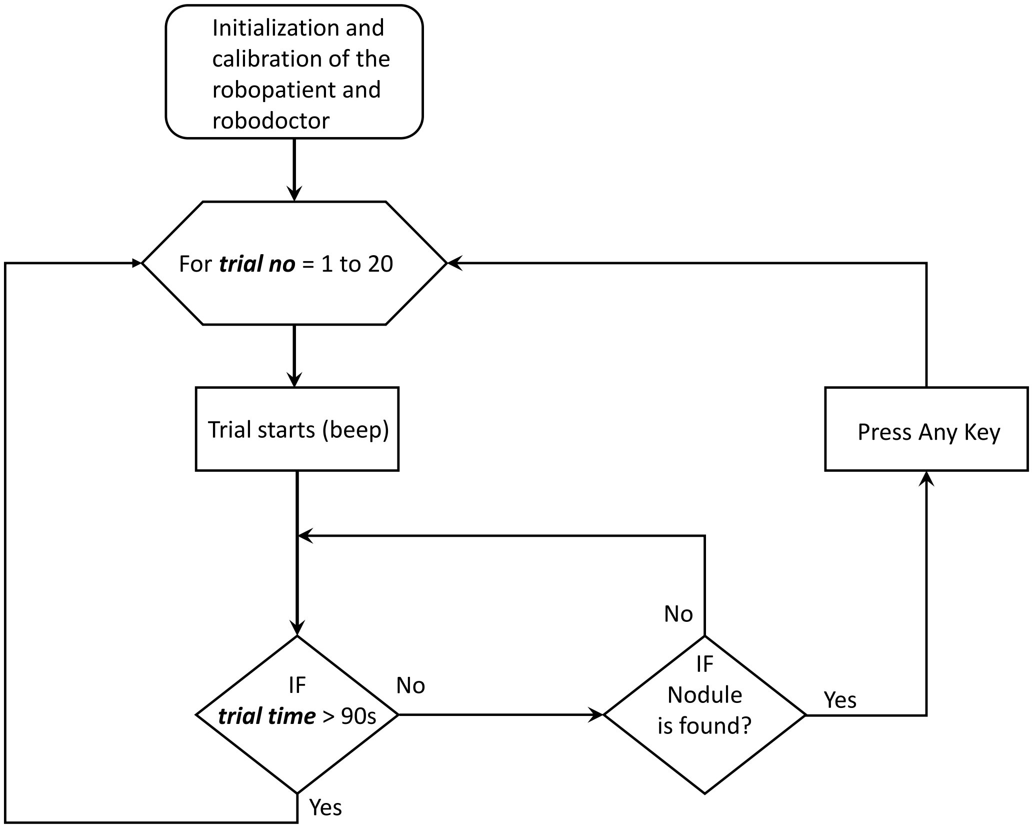

Figure S3. Flow chart of the experimental sequence used during two sessions. Each session (i.e: either with facial expression feedback or visual tactile map) consists of 20 trials. Each trial would last 90*s*.

actual nodule positions as .mat files for further analysis.

MatLAB 2020a was used for all statistical analysis in this study. Log transformed distribution of the differences between (1) medians of localization errors in the face and the visual tactile map was subjected to the Shapiro-Wilk test (using *swtest* function) for normality and then compared using one sample t-test (using *ttest* function). The Shapiro-Wilk test was also used to test the normality of the differences of (2) accuracy of localizing a hard nodule, (3) median nodule position estimation time and (4) number of unsuccessful trials between face mediated and visual tactile map approaches. Then, all these differences (i.e: (2), (3) and (4)) were compared using one sample t-tests separately. P values, value of the test statistic and degrees of freedom of the test are reported for all comparison tests, with $P < 0.05$ considered to be statistically significant. Effect sizes for individual participant were calculated based on Hedges' g using *meas*[1] toolbox.

S3 Description of Supplementary Videos

Movie S1: A video taken during a trial with the face mediated approach and visual tactile map feedback.

S4 References

1. Hentschke, H. hhentschke/measures-of-effect-size-toolbox. https://uk.mathworks.com/matlabcentral/fileexchange/ 32398-hhentschke-measures-of-effect-size-toolbox (2022).
